# Spatial Transcriptome Uncovers the Mouse Lung Architectures and Functions

**DOI:** 10.3389/fgene.2022.858808

**Published:** 2022-03-09

**Authors:** Yujia Jiang, Shijie Hao, Xi Chen, Mengnan Cheng, Jiangshan Xu, Chenghao Li, Huiwen Zheng, Giacomo Volpe, Ao Chen, Sha Liao, Chuanyu Liu, Longqi Liu, Xun Xu

**Affiliations:** ^1^ BGI College and Henan Institute of Medical and Pharmaceutical Sciences, Zhengzhou University, Zhengzhou, China; ^2^ BGI-Shenzhen, Shenzhen, China; ^3^ College of Life Sciences, University of Chinese Academy of Sciences, Beijing, China; ^4^ Hematology and Cell Therapy Unit, IRCCS Istituto Tumori “Giovanni Paolo II”, Bari, Italy

**Keywords:** spatial transcriptome, Stereo-seq, mouse lung, alveolarization, development

## 1 Introduction

Diseases leading to lung structural and functional disruption pose a major threat to human health (Tobin, 2005; Nathan et al., 2019; Podolanczuk et al., 2021). Mouse models represent a common choice to investigate physiological and pathological processes (Cheon and Orsulic, 2011; Benam et al., 2015; Walsh et al., 2017). To date, researches conducted on mouse lungs have been instrumental in dissecting developmental processes as well as pulmonary diseases, for example, studies on tumor-infiltrating myeloid cells in lung cancer (Zilionis et al., 2019), generation of pulmonary fibrosis (Wu et al., 2020), neonatal development (Scaffa et al., 2021) and injury repair (Leist et al., 2020). The recent development of novel state-of-the-art genomic technologies allows a deeper exploration of the lung functionally and structurally. A growing number of mouse-lung genomic datasets have been generated and have been paramount for illustrating fundamental transcriptomic changes at the tissue level (Angelidis et al., 2019; Vila Ellis et al., 2020) or revealing heterogeneity in resident lung cells (Angelidis et al., 2019; Gillich et al., 2020; Travaglini et al., 2020; Hurskainen et al., 2021) via bulk and single-cell RNA sequencing (scRNA-seq).

However, due to technical limitations of bulk and scRNA-seq, irreversible elimination of topological information hinders further analysis focusing on temporal-spatial dimensions (Lahnemann et al., 2020). In this regard, spatial transcriptome (ST) approaches, rising as the annual technology of Nature Methods in 2020 (Marx, 2021), can help to fill the knowledge gap between structure and function (Stahl et al., 2016; Lein et al., 2017; Moor and Itzkovitz, 2017). In this technique, spatio-temporal expression of genes is revealed by sequencing the mRNA captured *in situ*, with spatial coordinates labeled and translated (Close et al., 2021; Larsson et al., 2021; Zhuang, 2021). Using ST, researchers investigated the mechanisms of lung diseases and development at new latitudes, which is crucial for understanding the regulation of cell fate decisions and transitions of lung cells (Saviano et al., 2020; Liao et al., 2021). ST was first applied to mouse lung models by Boyd et al., in 2020, who reported the lethal immunopathology of the damage-responsive lung fibroblasts during severe influenza virus infection (Boyd et al., 2020). Furthermore, Xu found the location changes of T helper cells after immunization by integrating ST and scRNA-seq (Xu et al., 2021). Besides, ST was also employed to explore the mechanism that promotes lung tissue remodeling following respiratory viral infection (Beppu et al., 2021). The studies mentioned above have highlighted the importance of ST, in combination with multiple cutting-edge technologies, to gain basic and previously inaccessible information about lung biology and pathophysiology. However, most recent lung-related studies using ST are limited by a low spatial resolution (∼55–60 µm) and a restricted field of view (42.25 mm^2^), making the discrimination of different cells within the same capture spot challenging (Asp et al., 2020; Waylen et al., 2020). In addition, it is difficult to provide a detailed description of the subtle structures in the lung, such as bronchi, blood vessels, etc. Consequently, ST data of lungs with a resolution of approaching cellular-to-sub-cellular level are highly desired (Longo et al., 2021; Rao et al., 2021; Zhang et al., 2021).

Traditionally, mouse lung development can be subdivided into five main stages based on morphological changes: Embryonic (E9.5–E11.5), Pseudoglandular (E11.5–E16.5), Canalicular (E16.5–E17.5), Saccular (E17.5–P5) and Alveolar (postnatal (P) 5–P30), followed by maturation (P30 - adult) (Mujahid et al., 2013; Chao et al., 2015). Upon entering the maturation period, the basic structures of the mouse lung have developed and subsequently increase in size as the mouse grows (Schittny, 2017). It has been reported that the proximal lung matures more rapidly than the distal lung (Rawlins et al., 2007; Mund et al., 2008; Beauchemin et al., 2016; He et al., 2022). However, there is no research revealing the significant markers related to the spatial asynchronous development in the early maturation of mouse lung. Therefore, we selected P35 mouse lungs for spatiotemporal transcriptomic experiments to observe the spatial distribution of mature tissue cells within the mouse lung and dissect the spatial asynchronous development. For this study, we employed Stereo-seq (Chen et al., 2021), a novel ST approach with a 500 nm resolution that allows us to precisely detect mouse lungs’ structures and investigate the spatiotemporal transcriptomic change during lung development. Bioinformatic analysis on our dataset suggested the differences in the expression of several genes from proximal to distal lungs.

As far as we know, it is the first time to demonstrate such alternation via spatial gene mapping. In addition, our dataset provided strong support for further studies on the lung’s tissue structure and functional exercise and explored lung diseases’ mechanisms and treatment options. And the Stereo-seq data can be accessed and explored interactively on the website: https://db.cngb.org/stomics/datasets/STDS0000062?tab=explore.

## 2 Materials and Methods

### 2.1 Tissue Collection

For this study, we used a female adult mouse from a C57BL/6J strain, obtained from Guangdong Medical Laboratory Animal Center (https://www.gdmlac.com.cn/). The Institutional Review Board approved the use of laboratory mice on the Ethics Committee of BGI (Permit No. BGI-IR20210903001). Briefly, the mouse was sacrificed, and 1 × PBS (Thermo, CA, United States) was injected from the heart’s left ventricle to replace the blood until both lungs turned white. Then, the lung was inflated with the mixture of 50% optimal cutting temperature compound (OCT, SAKURA, Japan) and 50% 1 × PBS with 5% RNase inhibitor (RI, NEB, United States) dissolved (v/v) to complete the perfusion. After this, the lung was immediately isolated, snap-frozen with OCT in dry ice, and then stored in a −80°C refrigerator.

### 2.2 Tissue Cryosection, mRNA Capturing, and Sequencing

The Stereo-seq libraries were prepared as previously described (Chen et al., 2021). The OCT-frozen mouse lung was equilibrated in a Leica CM1950 cryostat (Leica Mikrosysteme Vertrieb GmbH, Wetzlar, Germany) at −17°C for 30 min. The lung was cryosectioned in 10 μm thickness. The first section was attached to a pathology-grade transparent microscope slide (CITOTEST, Jiangsu, China), while the following three successive sections were attached to the Stereo-seq chip surfaces. The microscope slide and chips were incubated at 37°C for 3 min and then transferred to methanol at −20°C for 30 min for fixation. The first section was stained with hematoxylin and eosin (H&E, Solarbio, Beijing, China). The Stereo-seq chips were dried at room temperature for 5 min, then permeabilized at 37°C using 0.1% PR Enzyme (BGI, Shenzhen, China) at pH 2.0 for 6 min. After that, the chips were washed twice using 0.1 × SSC with 5% RI v/v, and then dripped 100 μL reverse transcription (RT) mixture (5 μL RI, 5 μL RT Additive, 80 μL RT Reagent, 5 μL Reverse T Enzyme, 5 μL RT Oligo, BGI, Shenzhen, China) for 2 h at 42°C. Then the chips were washed twice with 0.1 × SSC. Tissues on the chip surfaces were then digested with the TR Buffer (BGI, Shenzhen, China) at 37°C for 30 min. The chips were washed twice with 0.1 × SSC and immersed in the cDNA Release Mix (10% cDNA Release Enzyme, 90% cDNA Release Buffer v/v, BGI, Shenzhen, China) at 55°C for 3 h to release the cDNA. The cDNA mixture was collected, and the chips were washed with nuclease-free H_2_O. The cDNA mixture and washing nuclease-free H_2_O were evenly mixed, and the new mixture was purified using the DNA clean beads (Vazyme, Nanjing, China) (0.8—fold to the volume of the new mix) to collect cDNAs in the target size. Finally, the cDNAs were amplified, fragmented, and cyclized to generate DNA nanoball (DNB) for sequencing on MGI DNBSEQ-T1 sequencer according to manufacturer protocol (MGI, Shenzhen, China) (Chen et al., 2021).

### 2.3 Stereo-Seq Raw Data Processing

The raw stereo-seq paired-end sequencing data was parsed to the coordinate identity (CID, read1 1st–25th base), unique molecular identifiers (UMI, read1 26th–35th base), and cDNA (whole reads 2) sequences at the first step, followed by the mapping of CID and pre-position-resolved CID whitelist by using the ST_BarcodeMap software (https://github.com/BGIResearch/ST_BarcodeMap) with a tolerance of 1-base mismatch. Next, the read pairs without valid CID sequencing data were removed. Then, according to the criteria, we filtered the read pairs with a low-quality UMI sequence, containing any N (undefined base) base or containing two bases with a quality score lower than 10 (Phred + 33). Next, the cDNA sequences with valid CID and UMI were aligned to the reference mouse genome (mm10) with the STAR software (Dobin et al., 2013). Moreover, we filtered out the alignments with a lower mapping quality (MAPQ < 10), annotated alignments on corresponding genes. Finally, we aggregated the raw bin 1 matrix into the final bin 50 matrix and assigned coordinates for each bin.

### 2.4 Spatially Variable Gene Detection and Spots Clustering

We applied the Hotspot software (v0.9.1) with the chosen model parameter “normal” to detect spatially variable genes (DeTomaso and Yosef, 2021). First, the variable genes were grouped into multiple groups with the compute_local_correlations function of Hotspot. Then, we calculated the gene module score for these detected gene groups, obtaining the final score matrix where each row represents a bin50 spots while each column represents a gene group. Finally, the bin50 spots were clustered into 10 clusters using the k-means function in the R language with the score matrix.

### 2.5 Cell Type Deconvolution With Single-Cell RNA-Seq Data

We used the RCTD software (v1.2.0) to resolve the cell composition in each bin 50 spot while the “doublet_mode” parameter was set as “multi” (Cable et al., 2021). The cell types with < 20 cells were pre-removed from the adoptive scRNA-seq data (Travaglini et al., 2020) because of the minimal cell number threshold in RCTD. Then we extracted the most likely cell composition in the “sub_weights” from the “result” slot to calculate the cell type correlation and count the overall relative cell number and visualization. Finally, the cell composition of each bin was visualized by scatterpie (v0.1.5) (https://github.com/GuangchuangYu/scatterpie) R package while we enlarged the spots Adventitial Fibroblast, Ciliated, and Vein for their concentrated location in the section.

### 2.6 Construction of the Spatial Regulatory Network

The regulating activity of transcription factors (TFs) was calculated by using SCENIC software (Aibar et al., 2017), implemented in pyscenic package (v0.11.2) (Van de Sande et al., 2020), in three steps. First, we calculated the co-expression genes for each TF using the grnboost2 software in step 1, followed by the mapping to the RcisTarget database for further validation of the regulative relation of the TFs and their relation co-expression genes. Finally, we ran the aucell command in pyscenic to calculate the regulatory activity of the TFs in each bin and visualized their spatial distribution.

## 3 Results

### 3.1 Stereo-Seq Data Quality Control

**FIGURE 1:**
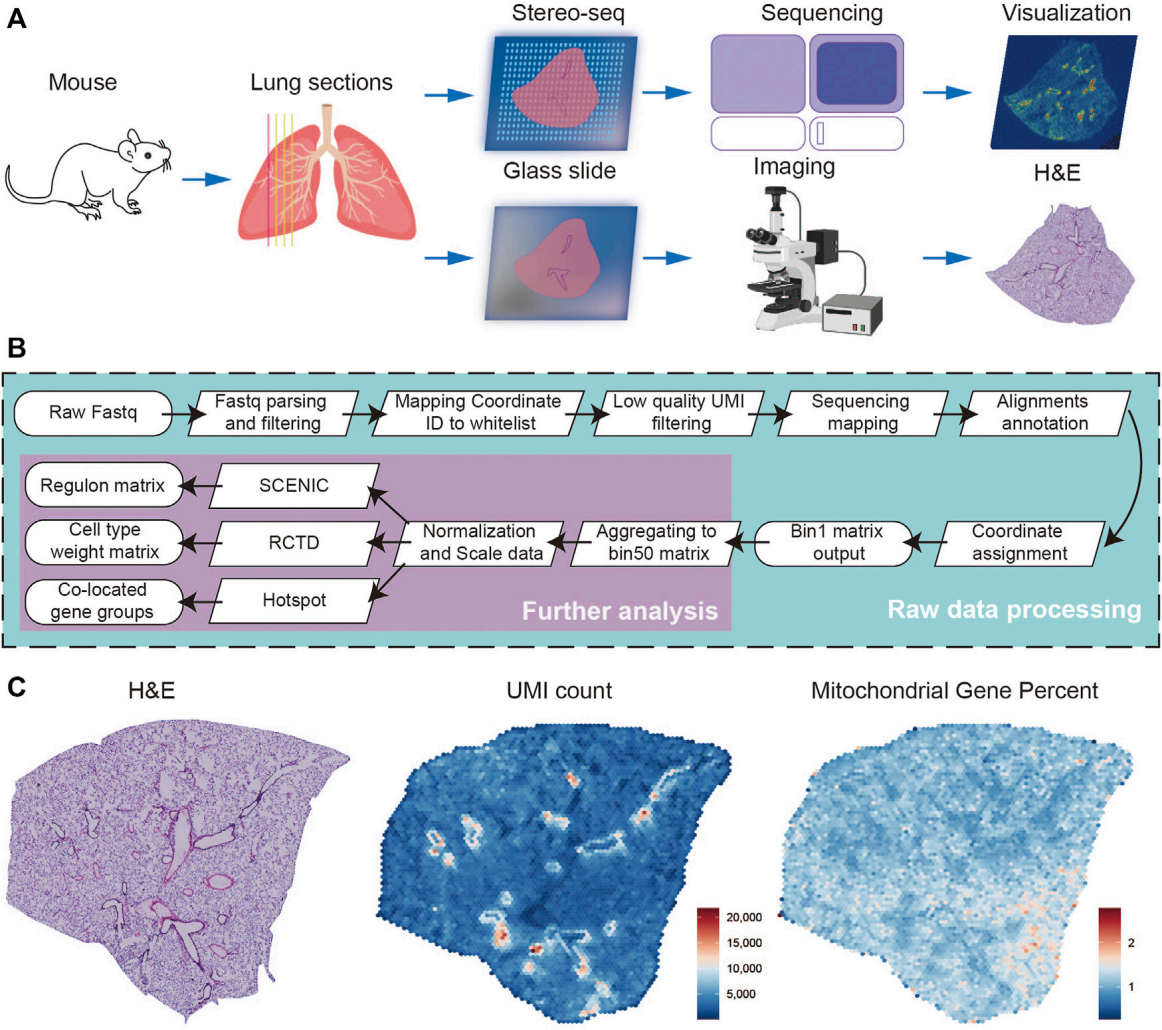
Overview of the experimental, data analysis workflow, and Stereo-seq data quality control. **(A)** Four lung sections from female mouse were collected, and the first was stained with H&E, the subsequent three for Stereo-seq experiments. **(B)** The analysis workflow for Stereo-seq datasets. **(C)** The H&E of the first section (left), the UMI (middle), and the percent of mitochondrial genes (right) spatial distribution patterns of the first Stereo-seq section.

A total of four successive frozen sections with a thickness of 10 μm were collected from a female adult mouse lung, the first of which was used for H&E staining and the subsequent three for Stereo-seq experiments (Figure 1A). Simultaneously, we systematically evaluated the quality of the Stereo-seq dataset based on the number of total reads, bases Q30, barcode mapping ratio, etc. (Supplementary Table S1). Approximately 4.77 G reads per section were generated, with the average bases Q30 of CID being 88.86%, the UMI Q30 was 84.28%, sequence Q30 was 88.61%, the average barcode mapping ratio was 61.79%, and the average reads mapping ratio was 79.87%. After further processing of alignments filtration, annotation, and matrix count, the bin1 Count Matrix was obtained. The bin1 represents a capture spot with a 220 nm diameter and a center-to-center distance of 500 nm. We combined the bin1 spots within a 50 × 50 square area as bin 50 and recognized them as the basic spot matrix for the subsequent analysis. The resolution of bin 50 is 25 μm (50 × 500 nm) (Figure 1B). To run quality control analysis on the Stereo-seq data of three sections, we characterized the overall distribution of UMI counts and the expression of mitochondrial genes separately, consistent with morphology photomicrographs displayed in H&E staining (Figure 1C). To assess the reproducibility of our data between different sections, we calculated the Coefficient of determination (R^2^), this being 0.9989 and 0.9980 for the Stereo-seq datasets in sections 2 and 1, and 3 and 1, respectively (Supplementary Figure S1).

### 3.2 Spatial Transcriptome Resolves the Lung Into Distinct Functional Regions

**FIGURE 2:**
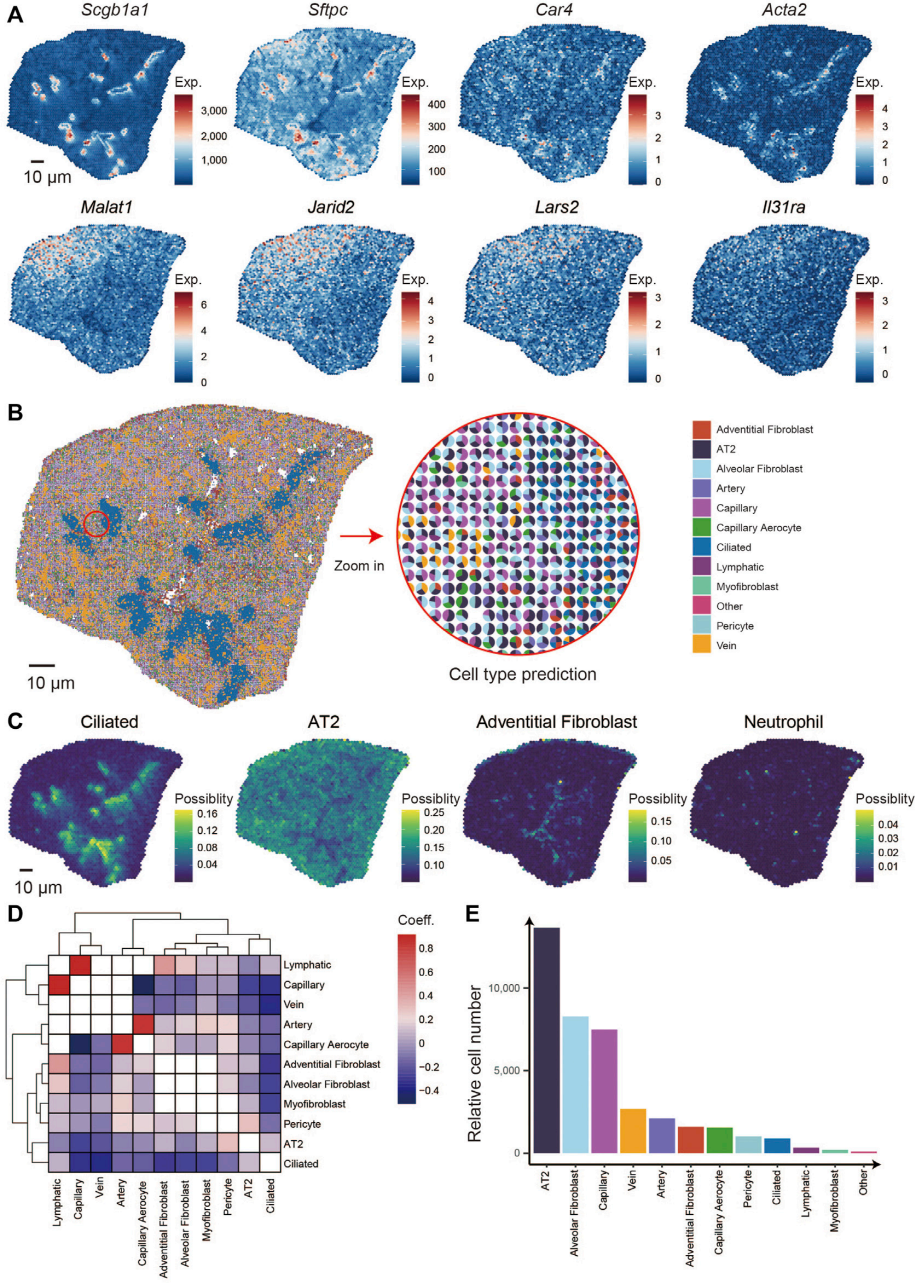
Identification of the specific pattern of genes in the lung and integrating analysis of scRNA-seq and Stereo-seq. **(A)** Spatial distribution of genes: *Scgb1a1*, *Sftpc*, *Car4*, *Acta2*, *Malat1*, *Jarid2*, *Lars2*, and *Il31ra*. **(B)** Spatial localization of multiple cell types in the lung section combines scRNA-seq and Stereo-seq (left) and the percentages of major cell types in each spot (right). **(C)** Spatial distribution of 4 cell types: ciliated, AT2, adventitial fibroblast, and neutrophil. **(D)** Spatial location correlation between different cell types. **(E)** The relative numbers of multiple cell types in the lung section.

We analyzed the spatial distribution of markers genes that are canonically expressed in bronchioli, alveoli, and immune cells, such as *Scgb1a1*, *Sftpc*, *Ager*, *Car4*, *Acta2*, *Ager*, *Dcn*, *Cdk8*, and *Foxj1* (Figure 2A). First, we calculated and clustered the spatially co-expressed genes by Hotspot software (v0.9.1) (DeTomaso and Yosef, 2021), thus leading to 17 groups that were reflected by typical expression patterns associated with given structures or cells in the lung (Supplementary Figure S2), for example, the expression of *Scgb1a1*, *Scgb3a2*, *Cyp2f2*, and *Hp* in group 1 corresponding to the bronchioli, group 2 with *Sftpc* and *Ager* for alveoli, group 7 with *Acta1* and *Acta2* for cartilage and group 8 with *Igkc*, *Jchain* and *Mzb1* for immune cells (Supplementary Figure S3A; Supplementary Table S2) (Han et al., 2018; Travaglini et al., 2020). Next, we integrated the module score of all gene groups. We then performed k-means clustering analysis to finally collapse the bin 50 spots into six defined clusters, obtaining a spatially-resolved pattern of multiple structures and cells (Supplementary Figure S3B). Finally, the percentages of all 6 clusters were counted within the section (Supplementary Figure S3C), and the correlations among the clusters were defined (Supplementary Figure S3D). Our analysis identified cluster 5 as highly enriched for Clara cells makers, those being *Scgb3a2*, *Scgb1a1*, *Cyp2f2*, and *Hp* (Supplementary Figure S3E; Supplementary Table S3). Considering that Clara cells are viewed as the main cells of lung bronchioli, it is safe to assume that the pattern observed in cluster 5 depicts the distribution of the bronchioli (Rawlins et al., 2009). Cluster 6 showed the highest correlation with cluster 5 and was distributed mainly around cluster 5, also being characterized by a similar expression pattern albeit at lower levels. The gradient distribution could result from a slight dispersion of lung bronchial cells.

Similarly, the spatial location of the intrapulmonary blood vessels was depicted by clusters 1, as characterized by the expression of *Myh6*, *Myl7*, and *Tnnt2*, which are typically expressed in the smooth muscle cells located around vessels (Thorolfsdottir et al., 2017; Cho et al., 2021). Cluster two and four displayed the highest correlation, were the most abundant within the analyzed section, and were marked by high expression of *Sftpc*, thus highlighting the distribution of alveoli within the mouse lung. *Malat1*, *Jarid2*, *Gm42428*, and *Cmm1* were observed at higher levels in cluster 4, indicating different alveolar states. The cluster three bins accounted for only a small percentage in the lung section and displayed high expression of canonical neutrophil genes such as *S100a9* and *S100a8*, indicating a population of immune resident cells (Han et al., 2018). To our knowledge, this is the first spatial transcriptome approach that allows a detailed description of the lung at the micrometer-scale resolution, and the data we obtained will be of great value in further understanding the spatial differences of lung development and diseases.

### 3.3 The Functional Gradient Along the Axis From the Proximal to the Distal Lung

Depending on the distance to the trachea, the top and bottom of the section were regarded as the distal and proximal lung, and clusters four and two were located at the distal and proximal areas, respectively (Supplementary Figure S3B) (Zepp et al., 2021). We calculated the differentially expressed genes (DEG) between Cluster two and Cluster four and found that *Malat1*, *Jarid2*, *Lars2*, *Il31ra*, *Camk1d*, *Cd44*, and others expressed higher in the distal lung. In contrast, *Scgb1a1*, *Scgb3s2*, *Cyp2f2*, *mt-Nd*4, *mt-Nd1*, *mt-Nd2*, and so on expressed higher at the proximal lung (Figure 2A; Supplementary Figure S4A). To reveal the particular functions of genes specially located in the different developmental areas, we performed the gene pathway enrichment analyses, which indicate that the proximal side is correlated with energy generation and transport such as ATP synthesis, and the distal with cell proliferation such as cell junction and assembly and positive regulation of binding (Supplementary Figure S4B). *Malat1*, a long non-coding RNA that is crucial for mouse lung development (Eissmann et al., 2012) and is overexpressed in a variety of lung cancers (Gutschner et al., 2013). *Jarid2* has been reported to control the cell cycle by regulating cyclin D1 in cardiac cells (Adhikari et al., 2019; Toyoda et al., 2003). *Lars2* is involved in mitochondrial function (Huang et al., 2021) and is enriched in endothelial cells of the brain and lung (Paik et al., 2020). *Il31ra* regulates allergen-induced lung inflammation (Neuper et al., 2021). *Cdk8* is a cycling-dependent kinase (Poss et al., 2013) that is overexpressed mainly in many tumor tissues and that accelerates the growth and division process of cancer cells (Supplementary Figure S3A) (Galbraith et al., 2017). Similarly, it has been reported that *Camk1d* was overexpressed when amplified in invasive breast carcinomas (Bergamaschi et al., 2008). Interestingly, *Cd44* has been reported to be expressed during the late saccular phase of human lung development at birth (Markasz et al., 2021).

The lung development of P35 mice is in a state of early maturation. Distributions of *Malat1*, *Jarid2*, *Lar2*, *Il31ra, Cdk8*, *Camkld* and *Cd44* indicated that the distal lung is more proliferative and less mature than the proximal during this developmental stage. It is evident that, at this particular stage, the development of the mouse lung is characterized by spatial asynchrony. Taken together, the spatial distribution of lung functional genes revealed by ST can aid our understanding of the function-structure relation of the lung structures.

### 3.4 Combinational Analysis of scRNA-Seq and Spatial Transcriptome

Thanks to the emergence of novel bioinformatic tools, it is now possible to integrate ST datasets with scRNA-seq data to obtain a deeper molecular insight while preserving spatial integrity, as shown in recent studies (Abdelaal et al., 2020; Kleshchevnikov et al., 2020; Kuppe et al., 2020; Fawkner-Corbett et al., 2021).

To further investigate the cellular spatial locations within the lung section, we comprehensively integrated of our Stereo-seq dataset with a previously published scRNA-seq dataset (Travaglini et al., 2020). We identified 11 main cell types within the analyzed lung section, including alveolar epithelial type 2 (AT2), artery, vein, ciliated, lymphatic, adventitial fibroblast, alveolar fibroblast, capillary, capillary aerocyte, myofibroblast, and pericyte (Figure 2B). Notably, the spatial distributions of different cell types, such as AT2, ciliated, alveolar fibroblast, and neutrophil, were consistent with the distribution of their marker genes expression as mentioned above (Figure 2C). And then, we calculated the correlation of spatial distribution between cell types in the entire lung section. Based on the results among the cell types, lymphatic and capillary were clustered together while the arterial cells clustered more closely with the capillary aerocyte cluster. This means that in the spatial distribution of this lung section, the lymphatic cell gathered the closest with capillary, the same as arterial cells and capillary aerocyte. The blank plots mean no correlation in any spatial location within those 2 cell types. The plots that belong to the cell type are also blank (Figure 2D). We calculated the percentage of each population of cells identified in each bin50 spot and observed that the AT2 was the most abundant, followed by alveolar fibroblast and capillary cells (Figure 2E) (Han et al., 2018).

Given the integration analyses are based on the resolution of bin 50, we visualized the spatial distribution of some marker genes at bin 1, bin 5, and bin 10 resolution, which is actually in the subcellular dimension. As a result, the gene expression pattern was roughly consistent with bin 50 and displayed more details (Supplementary Figure S5A). In addition, compared the distribution of genes and cells between Stereo-seq and a publicly available Visium dataset (Esquer et al., 2022), the highest resolution of Visium is 100 μm which can not accurately reveal the location of genes *in situ* (Supplementary Figure S5B).

Taken together, we have successfully integrated scRNA-seq and ST data, and those can be used for further and deeper multi-omics analysis.

### 3.5 Spatial Regulatory Activity of Transcription Factors in Lung

**FIGURE 3:**
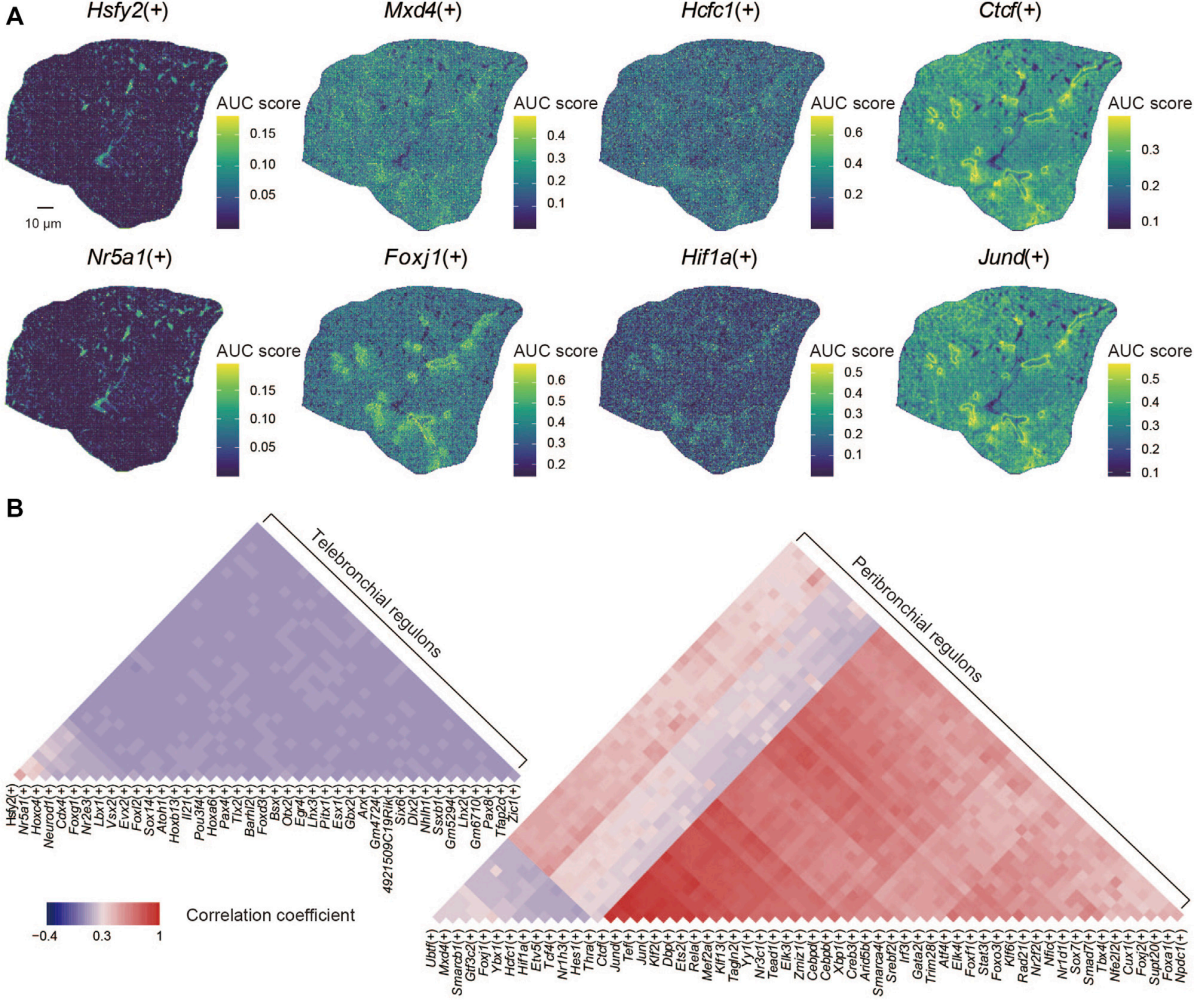
Spatial characteristics of regulons in the lung. **(A)** Spatial distribution of regulons: *Hsfy2*(+), *Nr5a1*(+), *Mxd4*(+), *Foxj1*(+), *Hcfc1*(+), *Hif1a*(+), *Ctcf*(+), and *Jund*(+). **(B)** The correlation coefficient of the spatial distribution of regulons.

Ultimately, we applied the SCENIC algorithm to our data for gene regulatory network reconstruction (Aibar et al., 2017). By doing so, we identified 99 regulons that displayed a distinct spatial distribution within the lung section, such as *Hsfy2*(+), *Nr5a1*(+), *Mxd4*(+), *Foxj1*(+), *Hcfc*(+), *Hif1a*(+), *Ctcf*(+), and *Jund*(+) (Figure 3A). We found that the spatial distribution of the regulatory activities of some regoluns remains consistent, such as *Ctcf*(+) and *Jund*(+). Next, we analyzed potential correlations between specific regulons with given lung structures (Figure 3B). We found that several regulon modules, including *Ctcf*(+), *Jund*(+), *Tef*(+), *Jun*(+), *Klf2*(+), *Dbp*(+), *Ets2*(+), *Foxj1*(+) were specifically enriched within the bronchioli. The regulon module with *Hcfc1*(+), *Hif1a*(+), *Etv5*(+), *Tcf4*(+), *Nr1h3*(+), *Hes1*(+) was instead preferentially located within alveoli, while the regulon module with *Hsfy2*(+), *Nr5a1*(+), *Hoxc4*(+), *Neurod1*(+), *Cdx4*(+), *Foxg1*(+), *Nr2e3*(+) was mainly distributed inside the alveoli according to the annotation of functional regions (Cusanovich et al., 2018). *Jund* has been reported to be involved in multiple mechanisms of cell fate regulation in the lung, including mediating transcriptional activation of angiotensinogen (Abdul-Hafez et al., 2009), inducing interleukin-6 (Eickelberg et al., 1999), and participating in lung carcinogenesis (Ruiz et al., 2021). *Foxj1* affects the differentiation and functional performance of ciliated cells distributed throughout the airway tree, including the trachea, lobar bronchi, and terminal bronchiole (You et al., 2004; Koay et al., 2021). *Hcfc1* drives cell-cycle gene expression as a transcriptional regulator, depending on heat-shock proteins 90 (Antonova et al., 2019). It was also shown that *Foxg1* is the main target gene of *MiR-378*, which promotes the cell proliferation of non-small cell lung cancer when *Foxg1* is inhibited (Ji et al., 2018). Similarly, *Tcf4* has been shown to play a role in developing lung cancer, for example, by promoting M2 polarization of macrophages (Sun and Xu, 2019). Our data can identify the spatial distribution of regulons in the mouse lung, providing fundamental insight for understanding its functions in development and diseases.

## 4 Conclusion

In summary, by applying the Stereo-seq, we provide an ST atlas of the developing murine female lung at an unprecedented resolution. The internal tissue structures of mouse lungs, such as bronchioli and alveoli, were well-exhibited, revealing the spatial localization of the regulons determined by SCENIC. Furthermore, our work developed a route for multi-modal-omics data integration by integrating of ST data with scRNA-seq. Our data can benefit several research areas in life sciences, such as lung developmental biology, disease research, tumor research, and more.

## Data Availability

The datasets presented in this study can be found in online repositories. The names of the repository/repositories and accession number(s) can be found below: https://www.ncbi.nlm.nih.gov/, PRJNA798781 https://db.cngb.org/, CNP0002590.

## Supplementary Material

Supplementary Figure 1 Stereo-seq dataset quality control of section 2 and section 3. **(A)** The UMI (left) and the percent of mitochondrial genes (middle) spatial distribution patterns of the Stereo-seq Section 2, and the Coefficient of determination (R^2^) of Section 2 and Section 1. **(B)** The UMI (left) and the percent of mitochondrial genes (middle) spatial distribution patterns of the Stereo-seq Section 3, and the Coefficient of determination (R^2^) of Section 3 and Section 1.

Supplementary Figure 2 Spatial distribution of the 17 hotspot gene groups.

Supplementary Figure 3 Identification of the pattern of genes and structures in the lung. **(A)** Spatial distribution of genes: *Ager*, *Dcn*, *Cdk8*, and *Foxj1*. **(B)** All groups to tissue section by clustering, to gain the spatial clustering. **(C)** Percentage of the content of each cluster in section. **(D)** Correlation of each cluster in the lung section. **(E)** Percentage expression and average expression of each cluster containing genes in the lung section.

Supplementary Figure 4 Identification of the differential express genes and functions in the proximal and distal lung. **(A)** The differential express genes in the proximal and distal lung. **(B)** The gene pathway enrichment analyses of the genes in the proximal and distal lung.

Supplementary Figure 5 The distribution of genes and cells in Stereo-seq and Visium. **(A)** The distribution of *Acta2*,*Tmem100*, *Myh6, and Jarid2* in bin1, bin5, and bin10 of Stereo-seq. **(B)** The distribution of Ciliated, Adventitial Fibroblast, and AT2 of Visum.

Supplementary Table 1 Sequencing dataset quality control of three Stereo-seq sections.

Supplementary Table 2 List of genes in groups formed by identical expression patterns.

Supplementary Table 3 Markers and annotation for each cluster in the mouse lung section.
