# Supplementary figures and images for: Spatial Transcriptome Uncovers the Mouse Lung Architectures and Functions
Source: Front Genet. 2022 Mar 9;13:858808. doi: 10.3389/fgene.2022.858808 (PMC8982079; doi:10.3389/fgene.2022.858808)

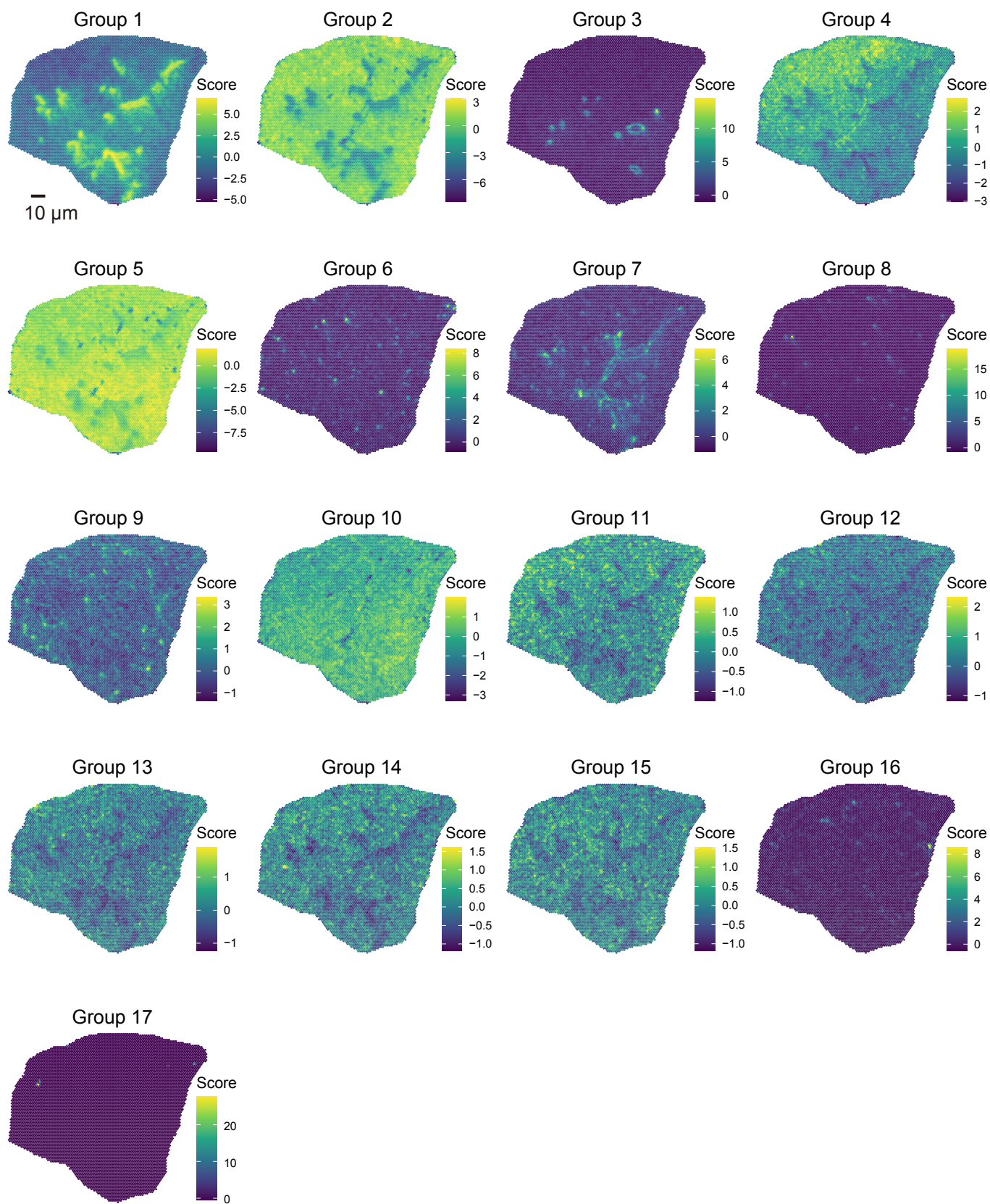

Supplementary Figure 2

Supplement: Supplementary file 1 [file DataSheet2.PDF]

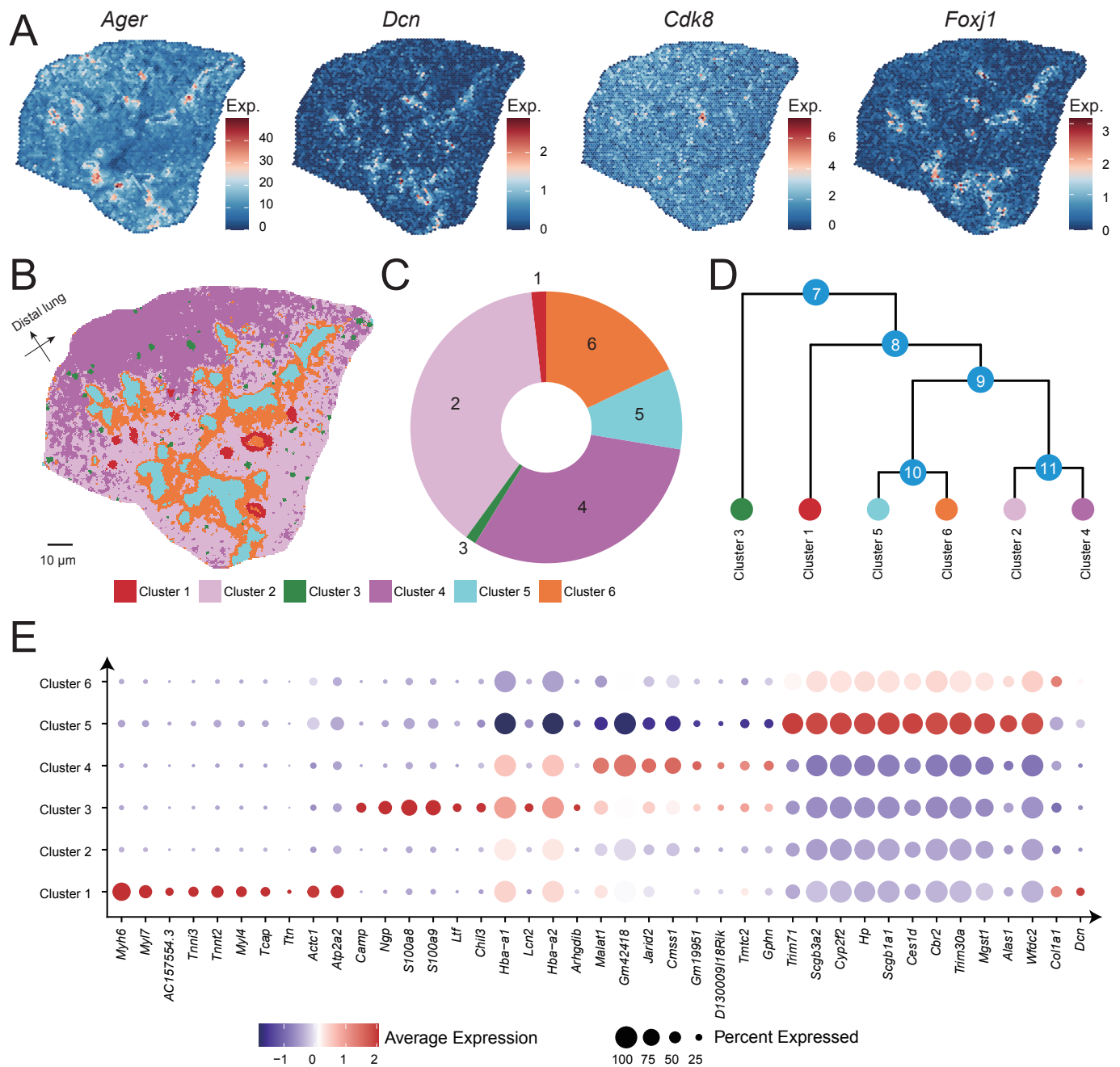

Supplementary Figure 3

Supplement: Supplementary file 5 [file DataSheet3.PDF]

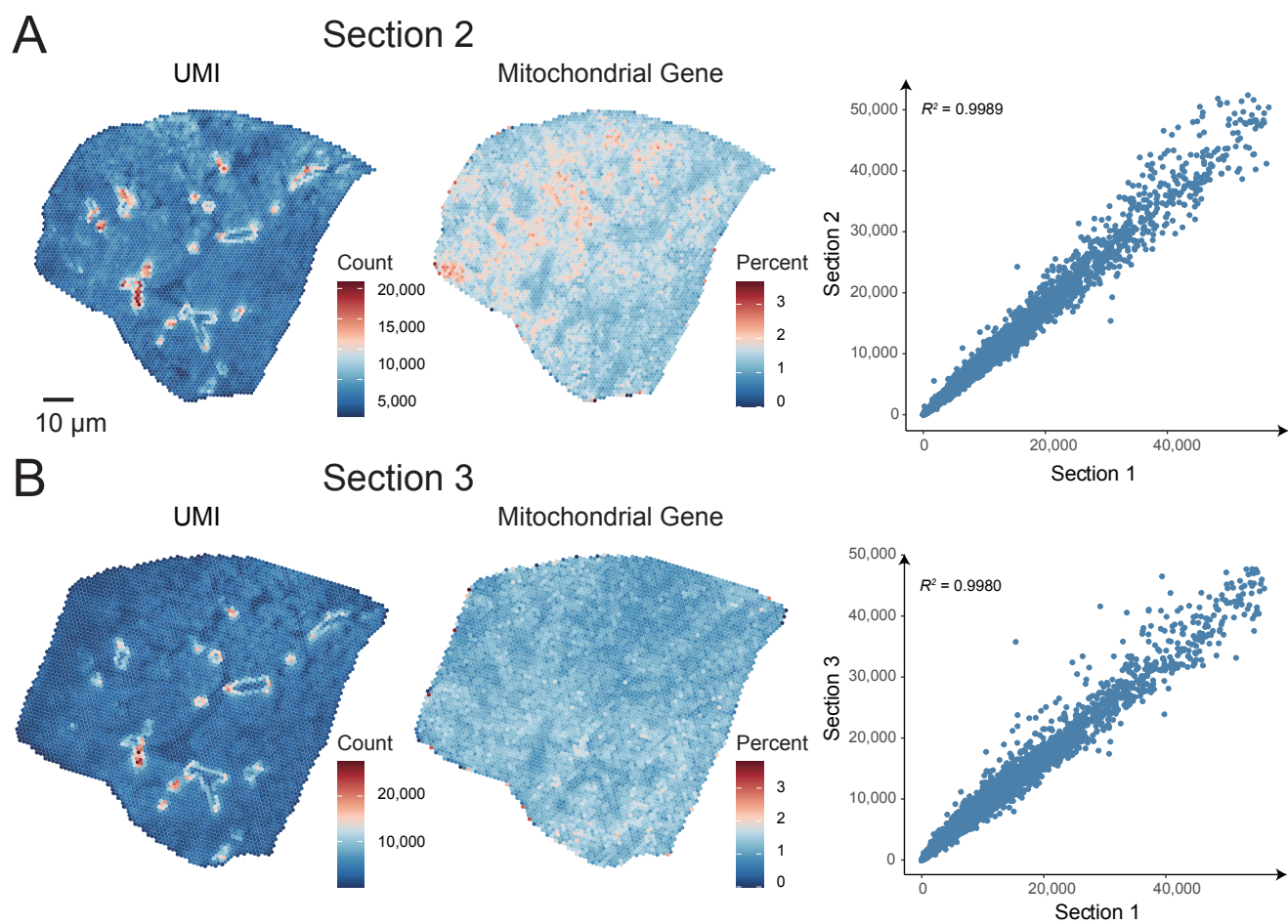

Supplementary Figure 1

Supplement: Supplementary file 7 [file DataSheet1.PDF]

**A***Acta2* bin1*Acta2* bin5*Acta2* bin10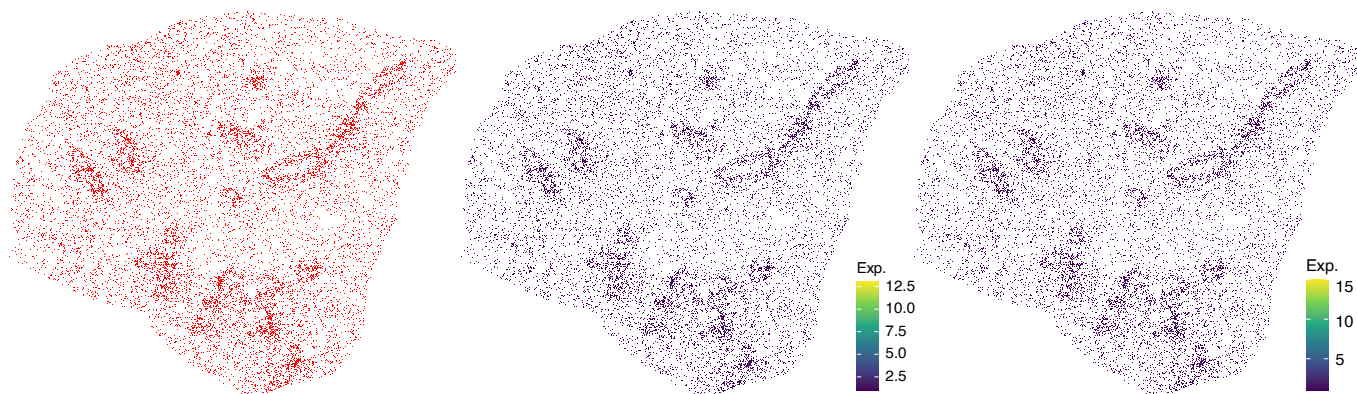**B**

Ciliated

Adventitial Fibroblast

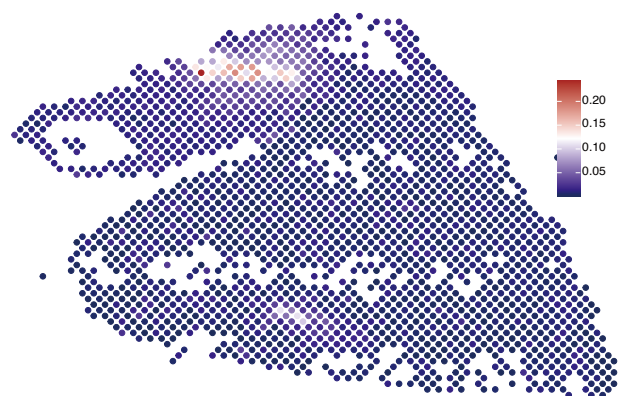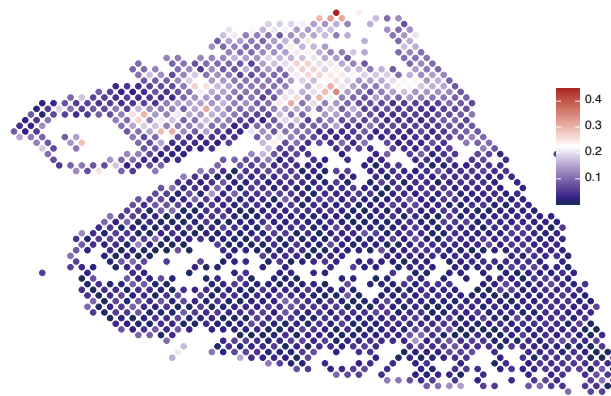

Alveolar Epithelial Type 2

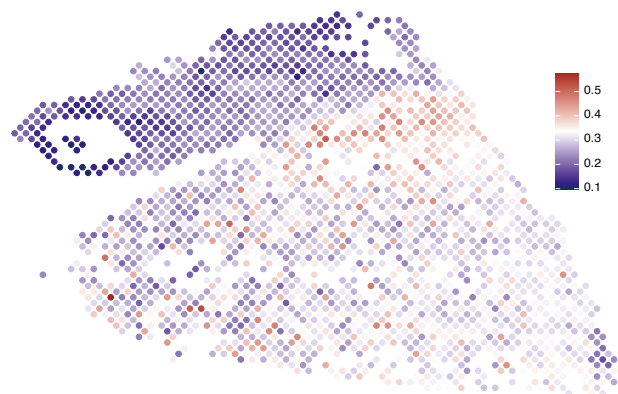

Supplement: Supplementary file 8 [file DataSheet5.PDF]
