# Supplementary material for: Spatial Transcriptome Uncovers the Mouse Lung Architectures and Functions
Source: Front Genet. 2022 Mar 9;13:858808. doi: 10.3389/fgene.2022.858808 (PMC8982079; doi:10.3389/fgene.2022.858808)
Supplement: Supplementary file 3 [file DataSheet4.PDF]

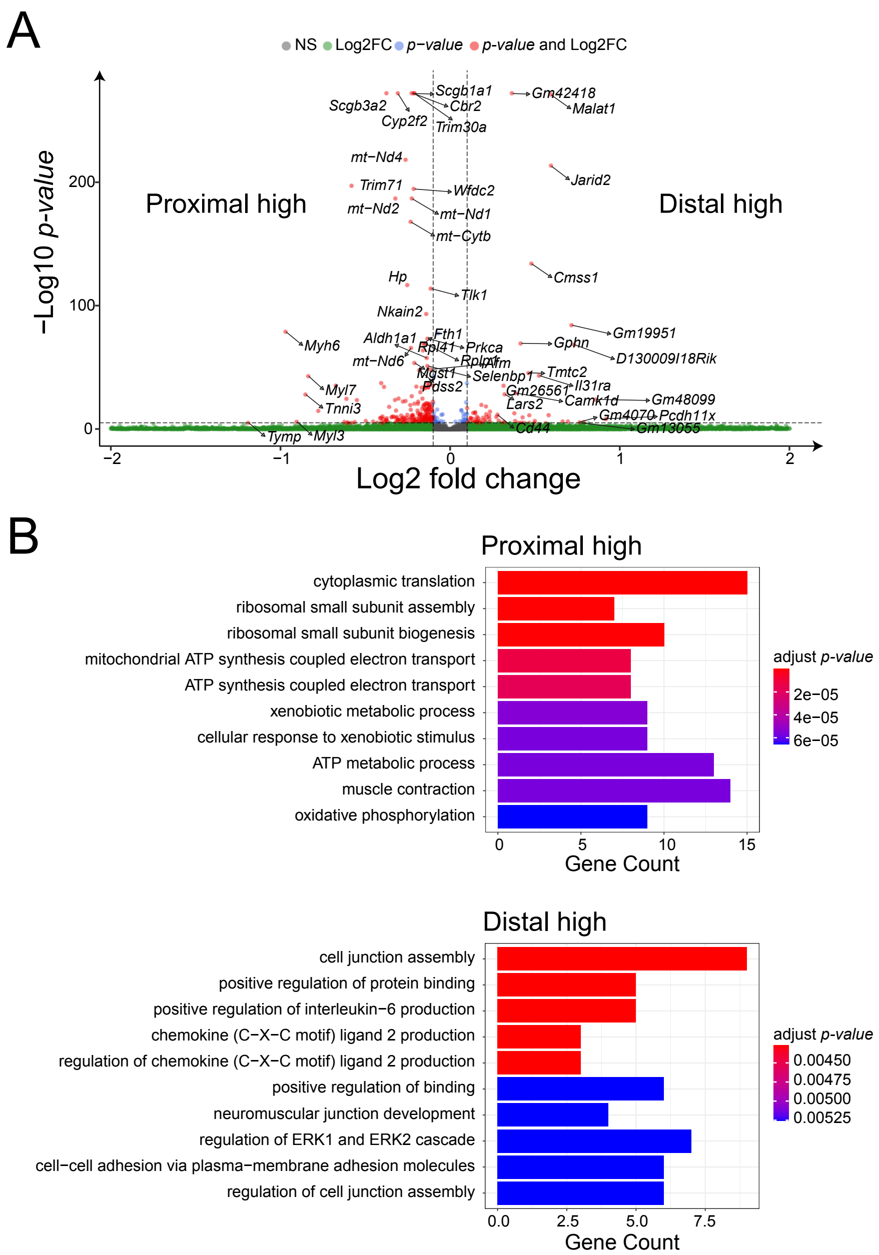

Supplementary Figure 4
